# Age-Dependent Ocular Dominance Plasticity in Adult Mice

**DOI:** 10.1371/journal.pone.0003120

**Published:** 2008-09-01

**Authors:** Konrad Lehmann, Siegrid Löwel

**Affiliations:** Institut für Allgemeine Zoologie und Tierphysiologie, Friedrich-Schiller-Universität Jena, Jena, Germany; University of Southern California, United States of America

## Abstract

**Background:**

Short monocular deprivation (4 days) induces a shift in the ocular dominance of binocular neurons in the juvenile mouse visual cortex but is ineffective in adults. Recently, it has been shown that an ocular dominance shift can still be elicited in young adults (around 90 days of age) by longer periods of deprivation (7 days). Whether the same is true also for fully mature animals is not yet known.

**Methodology/Principal Findings:**

We therefore studied the effects of different periods of monocular deprivation (4, 7, 14 days) on ocular dominance in C57Bl/6 mice of different ages (25 days, 90–100 days, 109–158 days, 208–230 days) using optical imaging of intrinsic signals. In addition, we used a virtual optomotor system to monitor visual acuity of the open eye in the same animals during deprivation. We observed that ocular dominance plasticity after 7 days of monocular deprivation was pronounced in young adult mice (90–100 days) but significantly weaker already in the next age group (109–158 days). In animals older than 208 days, ocular dominance plasticity was absent even after 14 days of monocular deprivation. Visual acuity of the open eye increased in all age groups, but this interocular plasticity also declined with age, although to a much lesser degree than the optically detected ocular dominance shift.

**Conclusions/Significance:**

These data indicate that there is an age-dependence of both ocular dominance plasticity and the enhancement of vision after monocular deprivation in mice: ocular dominance plasticity in binocular visual cortex is most pronounced in young animals, reduced but present in adolescence and absent in fully mature animals older than 110 days of age. Mice are thus not basically different in ocular dominance plasticity from cats and monkeys which is an absolutely essential prerequisite for their use as valid model systems of human visual disorders.

## Introduction

Ocular dominance plasticity induced by monocular eyelid suture is one of the best studied models of experience-dependent cortical plasticity [1]. Neurons in the binocular part of the visual cortex respond to inputs from both eyes, but are dominated by the contralateral eye in rodents [2], [3]. Brief monocular deprivation in early postnatal life induces a shift in the ocular dominance of binocular neurons towards the open eye [4], [5]. In mice, the peak of the critical period for ocular dominance plasticity lies between postnatal days 25 and 30, when four days of monocular deprivation are sufficient to make binocular cortical neurons equally responsive to both eyes [5].

Only recently it has been observed that ocular dominance plasticity can also be observed in young adult mice, although after longer deprivation periods (6–7 days) [6]–[8]. However, these reports of “adult” ocular dominance plasticity so far appear somewhat contradictory. While some groups find significant plasticity using visual evoked potential recordings, optical imaging or immediate early gene induction [6]–[10], others hardly detect it at all [11] or fail to find any adult plasticity using single unit recordings [12], [13].

While methods are certainly an issue, we also noticed that most studies investigated animals at 60–90 days of age, which is late adolescence in mice. So far only few groups have studied fully adult mice, i.e. animals >100 days old, and found little [11] or no [13] ocular dominance plasticity after seven days of monocular deprivation. We therefore wondered whether so-called “adult” ocular dominance plasticity in mice might be age-dependent, and whether it would still be detectable in fully grown animals >4 months of age.

As a means of assessing visual cortical plasticity we have used two different techniques: (i) optical imaging of intrinsic signals to visualize the ocular dominance shift of neurons after monocular deprivation [14], [15] and (ii) a virtual optomotor system [16] to monitor the enhancement of vision in the open eye after monocular deprivation in the same animals. Here, we show that there is an age-dependence of both ocular dominance plasticity in the visual cortex and the increase in visual acuity after monocular deprivation: while in mice younger than 100 days, monocular deprivation induced a significant ocular dominance shift, such a shift was absent in animals aged 110–230 days even after longer deprivation times (up to 14 days), and the enhancement of vision of the nondeprived eye also significantly declined in animals older than 110 days.

## Materials and Methods

### Animals and rearing conditions

Male C57BL/6 mice were raised in standard cages on a 12 h light/dark cycle, with food and water available *ad libitum*. Animals were reared in sibling groups and isolated at four months of age. All experimental procedures were approved by the local government under the registration number 02-015/06.

### Monocular deprivation

For probing visual cortical plasticity, we monocularly deprived mice according to published protocols [5], [14]. In all cases, the right eyes were sutured shut. Animals were checked daily to make sure that the eyes remained closed; animals in which the eye was not completely closed were excluded from the experiments.

Animals of four different age groups were used in the present experiments: 1. PD25 = mice monocularly deprived during the critical period of early postnatal development (deprivation onset between postnatal days 24–26). 2. PD95 = young adult mice (deprivation onset between postnatal days 90–100). 3. PD130 = fully adult mice (deprivation onset between postnatal days 109–158). 4. PD215 = mature mice (deprivation onset between postnatal days 208–230).

### Visual acuity and contrast sensitivity

Visual acuity was assessed using the recently developed virtual optomotor system [16]. Briefly, freely moving animals are exposed to moving sine wave gratings of various spatial frequencies and contrasts and will reflexively track the gratings by head movements as long as they can see the gratings. Spatial frequency at full contrast and contrast at six different spatial frequencies were varied by the experimenter until the threshold of tracking was determined.

### Surgical preparations for optical imaging

After initial anaesthesia with 2% halothane in 1:1 O_2_/N_2_O mixture, the animals received an intraperitoneal injection of 50 mg/kg pentobarbital, supplemented by chlorprothixene (0.2 mg/mouse, i.m.), atropine (0,3 mg, s.c.) and dexamethasone (0.2 mg/mouse, s.c.). A tracheotomy was performed and the animals were placed in a stereotaxic apparatus. In addition, lidocaine (2% xylocaine jelly) was applied locally to all incisions. Body temperature was maintained at 37°C and the ECG was monitored throughout the experiment. Anaesthesia was maintained with 0.6–0.8% halothane in a mixture of 1:1 O_2_/N_2_O applied through the tracheal tube. In some experiments, 1.2 mg/kg urethane was used for general anaesthesia. A craniotomy was made over the hemisphere contralateral to the deprived eye in monocularly deprived animals. The exposed area was covered by agarose (2.5%) and a glass coverslip.

Mouse visual cortical responses were recorded using the imaging method developed by Kalatsky and Stryker [15] and optimized for the assessment of ocular dominance plasticity by Cang et al. [14]. Briefly, optical images of cortical intrinsic signals were obtained using a Dalsa 1M30 CCD camera (Dalsa, Waterloo, Canada) controlled by custom software. Using a 135×50 mm tandem lens configuration (Nikon, Inc., Melville, NY), we imaged a cortical area of 4.6×4.6 mm^2^. The surface vascular pattern and intrinsic signal images were visualized with illumination wavelengths set by a green (550±3 nm) or red (610±3 nm) interference filter, respectively. Frames were acquired at a rate of 30 Hz, temporally binned to 7.5 Hz and stored as 512×512 pixel images after spatial binning of the camera image. A high refresh rate monitor (Hitachi Accuvue HM 4921-D) was placed in front of the animal (at 25 cm distance) to display the visual stimuli: horizontal bars drifted at a spatial frequency of 0.0125 cycles/degree (cyc/deg), and a temporal frequency of 0.125 Hz. Visual stimulation was restricted to the binocular visual field of the recorded hemisphere (−5° to +15° azimuth).

### Data analysis

Maps were calculated from the acquired frames by Fourier analysis to extract the signal at the stimulation frequency using custom software [15]. While the phase component of the signal is used for the calculation of retinotopy, the amplitude component represents the intensity of neuronal activation and can be used to calculate ocular dominance (for details see [14]): an ocular dominance score of each pixel in the binocularly active region was calculated as (C−I)/(C+I), with C and I representing the raw response magnitudes of each pixel to the contralateral and ipsilateral eye, respectively. An ocular dominance index (ODI) was then computed as the average of the ocular dominance scores of all responsive pixels. Consequently, ODI ranges from −1 to 1, negative values representing ipsilateral, positive values contralateral bias.

We calculated ODIs from blocks of four runs in which the averaged map for each eye had at least a response magnitude of 1×10^−4^. Typically, we obtained at least five ODIs per animal; experiments with less than three ODIs were discarded. The ODIs of one animal were averaged for statistical comparisons between ages and deprivation conditions.

### Statistical analysis

All inter-group comparisons were done by two-way ANOVA with age and deprivation duration as the independent variables. For the behavioural data, days after monocular deprivation and spatial frequencies were defined as repeated measurement factors. Post-hoc tests were carried out with Bonferroni correction. The levels of significance were set as *: p<0.05; **: p<0.01; ***: p<0.001. Data are represented as means±s.e.m.

## Results

### Ocular dominance plasticity after monocular deprivation declines with age

**Figure 1:**
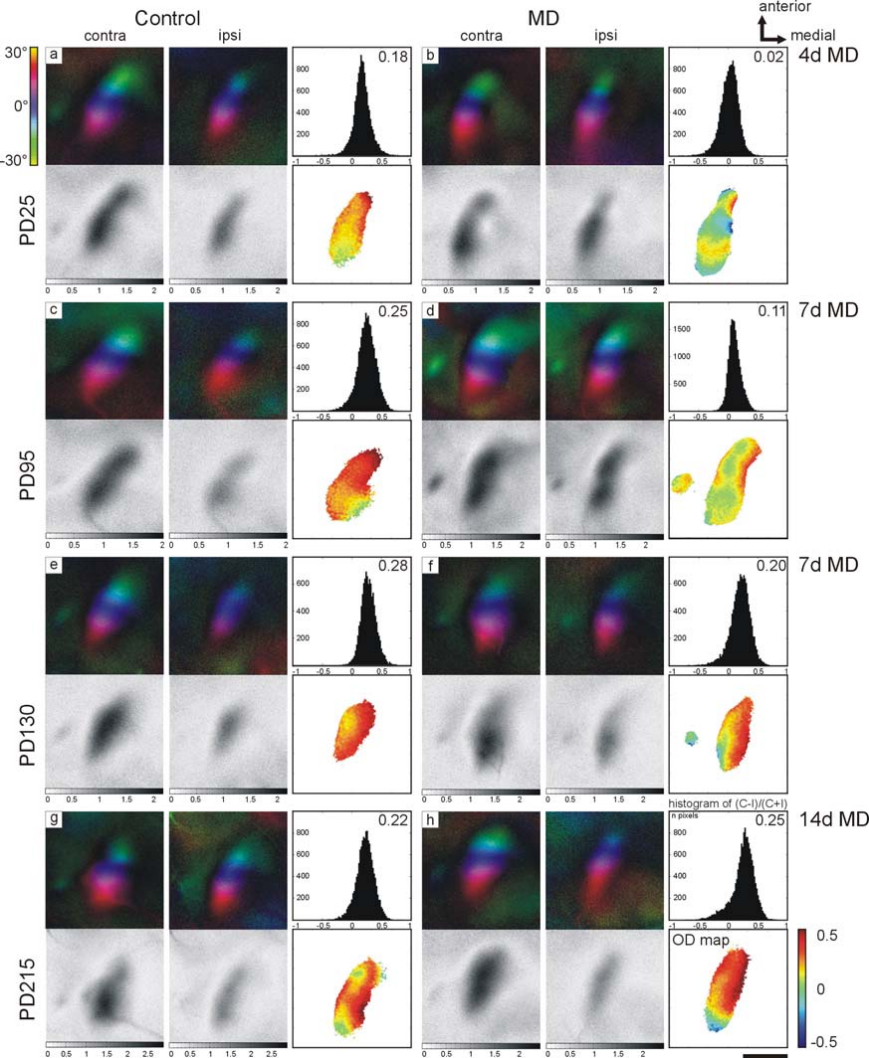
Ocular dominance plasticity in mouse visual cortex is age-dependent. Representative experiments of animals in all four age groups studied (PD25, PD95, PD130 and PD215) are displayed. Optical imaging maps of responses to the ipsi- and contralateral eye in the binocular region of mouse visual cortex in both control animals (left column: a, c, e, g) and monocularly deprived animals (right column: b, d, f, h) are shown. Both colour-coded polar maps of retinotopy (top) and grey-scale coded response magnitude maps (below) are illustrated. For each experiment, the histogram of ocular dominance scores, the average ocular dominance index (ODI) and the corresponding 2-D ocular dominance maps (ODI values colour-coded according to the scheme shown in the lower right corner of the figure: blue represents negative, red positive values) is included. Note that in control animals of all ages, activity patches evoked by the stimulation of the contralateral eye were consistently darker than those after stimulation of the ipsilateral eye (a, c, e, g) and that 2-D ocular dominance maps are red and yellow indicating contralateral dominance. In contrast, monocular deprivation for 4 days in PD25 animals (b) or for 7 days in PD95 animals (d) induced a significant ocular dominance shift so that the response magnitude maps of both ipsi- (open) and contralateral (deprived) eye are now equally dark, the histograms of ocular dominance scores shift to the left (compare a to b and c to d) and colder colours prevail in the 2-D ocular dominance maps. In the two older animal groups, PD130 and PD215 mice, monocular deprivation for 7 days (f) or 14 days (h) fail to induce ocular dominance shifts and both histograms of ocular dominance scores and 2-D ocular dominance maps are similar to control animals (compare e to f and g to h). The scale bar is 1 mm and applies to all panels showing maps. Abbreviations: MD = monocular deprivation, OD = ocular dominance, contra = contralateral eye, ipsi = ipsilateral eye.

We studied ocular dominance plasticity in the visual cortex using mice of four different age groups: PD25, PD95, PD130 and PD215 (see Methods for details). Using optical imaging of intrinsic signals [14], we compared the response amplitudes in the binocular region of visual cortex after stimulation of the ipsi- and contralateral eye in both normally raised animals and in animals after various periods of monocular deprivation (Fig. 1).

**Figure 2:**
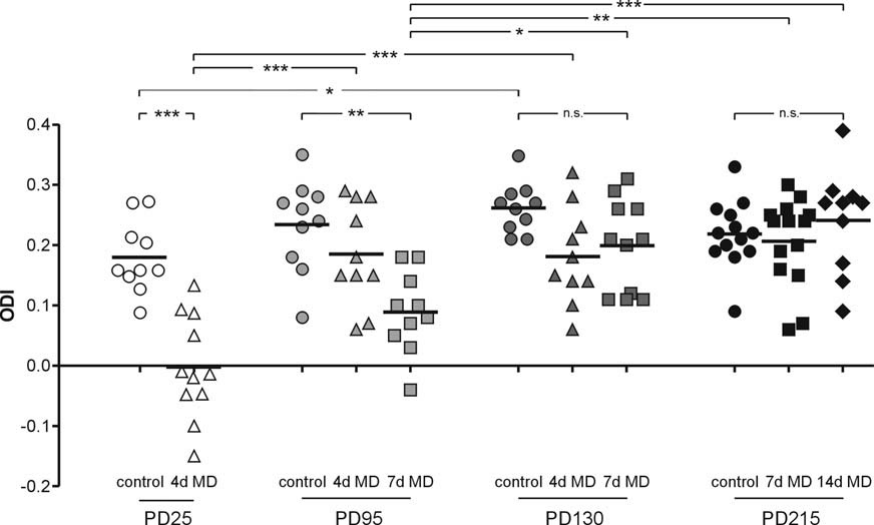
Decline of ocular dominance plasticity after monocular deprivation in animals older than 110 days of age. ODIs of control and monocularly deprived animals of all age groups. A positive ODI indicates dominance of the contralateral eye, a negative ODI ipsilateral dominance. Symbols represent ODI values of individual animals; means are marked by the thick horizontal lines. Circles represent values obtained from control animals, triangles, squares and diamonds values of animals after 4 days (4d), 7 days (7d) and 14 days (14d) monocular deprivation, respectively. Note that four days of monocular deprivation in PD25 and 7 days of monocular deprivation in PD95 animals induced a significant ocular dominance shift towards the open eye (p<0.001 and p<0.01, respectively, Bonferroni-corrected t-test). In both PD130 and PD215 animals, monocular deprivation has no such effect. In addition, the ODI of PD95 animals after 7 days of monocular deprivation was significantly different from the ODIs of PD130 and PD215 animals after the same deprivation period (p<0.05 and p<0.01, respectively, Bonferroni-corrected t-test).

In control animals of all ages, activity patches of the contralateral eye were always darker than those of the ipsilateral eye, reflecting the dominance of the contralateral eye in the binocular region of rodent visual cortex (Figs. 1a, c, e, g, left column). Two-dimensional maps of the ocular dominance scores (the ocular dominance map) in the binocular region of visual cortex are displayed for all experiments in Figure 1. In addition, ocular dominance index (ODI) histograms are illustrated. Control animals of all ages had average ODIs of around 0.2 (PD25: 0.18±0.019; PD95: 0.23±0.024; PD130: 0.26±0.013, all n = 10; PD215: 0.22±0.016, n = 13, see Fig. 2), and the ocular dominance maps showed warm colours indicating a clear contralateral dominance (Figs. 1a, c, e, g; right column). Comparing the ODIs of all age groups, there was a significant influence of age (F_3,39_ = 3.307, p<0.05, ANOVA), with a group difference between PD25 and PD130 (p<0.05, Bonferroni post-hoc). Control ODIs of PD95 and PD215 were not statistically different from any other group (p>0.2 in all comparisons).

To investigate the influence of monocular deprivation on ocular dominance, we performed monocular deprivation for 4, 7 or 14 days. The effects of the longest periods of monocular deprivation in each age group are displayed in Figure 1 (Figs. 1b, d, f, h). Four days of monocular deprivation during the critical period strongly shifted the ocular dominance towards the ipsilateral (open) eye and optically recorded maps of the contralateral and ipsilateral eye were almost equally strong (Fig. 1b). The average ODI was 0±0.026 (n = 11, p<0.001 vs. control). In young adult animals (PD95), 7 days of monocular deprivation was necessary to induce a significant ocular dominance shift (Figs. 1d and 2) with an average ODI of 0.09±0.021 (n = 10, p<0.01). While four days of monocular deprivation decreased the average ODI in this age group compared to controls (0.19±0.027, n = 10; Fig. 2), the difference was statistically not significant (p>0.5).

Interestingly, in all older age groups (PD130 and PD215), 7 or even 14 days of monocular deprivation had no significant effect on the ocular dominance and average ODIs were statistically indifferent from control values (Figs. 1f, h and 2).

Statistical analysis confirmed these results. Two-way ANOVA indicated that monocular deprivation efficiently altered ODIs (F_3,108_ = 13.135, p<0.001). Age had an influence on ocular dominance (F_3,108_ = 14.931, p<0.001), and there was an interaction of age and monocular deprivation (F_4,108_ = 6.339, p<0.001), showing that monocular deprivation altered ocular dominance differently in the four age groups. This relationship was further investigated by Bonferroni-corrected pair-wise comparisons using t-tests. Four days of monocular deprivation were sufficient to reduce the ODI of PD25 mice from 0.18±0.019 (n = 10) to 0±0.026 (n = 11, p<0.001 vs. control). In contrast, 4 days of monocular deprivation were insufficient to induce a significant ocular dominance shift in both PD95 and PD130 animals. Thus, the ODI of PD25 animals after 4 days of monocular deprivation was significantly different from the two older age groups in which a 4 day monocular deprivation was performed (p<0.001).

In PD95 animals, seven days of monocular deprivation were necessary to significantly shift the ocular dominance (p<0.01; Figs. 1d and 2). In contrast, in animals just one month older (PD130), monocular deprivation of both 4 days and 7 days failed to induce significant ocular dominance shifts (4 days monocular deprivation: 0.18±0.025, n = 10; 7 days monocular deprivation: 0.2±0.023, n = 11; p = 0.13 and 0.43, Figs. 1f and 2). Intriguingly, after 7 days of monocular deprivation, PD95 animals differed significantly from PD130 and PD215 animals (p<0.05 and p<0.01), emphasizing the rapid decline of ocular dominance plasticity in animals >100 days old.

In fully mature animals (>7 months, PD215), 7 days of monocular deprivation did also not reduce the ODI (0.21±0.018, n = 14, p = 1, Fig. 2). We therefore tried a longer deprivation period (14 days), but even that did not result in a significant ocular dominance shift (0.24±0.027, n = 10, p = 1; Fig. 1h).

### Influence of anaesthetic on ocular dominance plasticity

Since several recent studies have provided evidence that some anaesthetics - in particular barbiturates - might mask ocular dominance plasticity in adult animals [9], [11], we repeated some of our experiments in PD215 animals using urethane anaesthesia. In these experiments, 7 days of monocular deprivation did not induce a significant ocular dominance shift compared to controls (controls: 0.22±0.02, n = 3; 7 days monocular deprivation: 0.25±0.004, n = 4; p = 0.19, t-test). Since the ODIs of both normally raised mice and 7 days monocular deprivation in PD215 animals were indistinguishable from values obtained in halothane-anaesthetized animals (control: p = 0.86, 7 days monocular deprivation: p = 0.17, t-test), we have pooled data from both anaesthesia-regimes for the final statistical analyses and data presentation.

### Enhancement of vision after monocular deprivation declines with age

**Figure 3:**
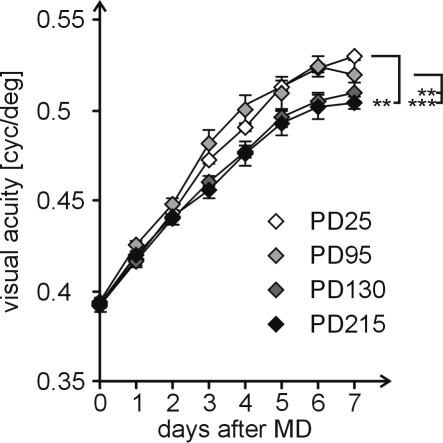
Enhancement of vision after monocular deprivation declines with age. Visual acuity of the animals was analyzed using a virtual optomotor system (Prusky et al., 2004). During 7 days of monocular deprivation and daily testing, spatial frequency selectivity of the optokinetic response of the nondeprived (open) eye increased from 0.39 cyc/deg to approximately 0.52 cyc/deg. While interocular plasticity was present in all analyzed age groups, it was significantly stronger in PD95 compared to PD130 and PD215 animals (p<0.01 and p<0.001), and in PD25 animals compared to PD215 animals (p<0.01, Bonferroni post hoc).

As recently reported, monocular deprivation induces an enhancement of the optokinetic response of the nondeprived eye in adult mice [17]. In our experiments, baseline visual acuity was 0.39 cycles/degree (cyc/deg), with no significant differences between age groups (F_3,74_ = 1.876, p = 0.14, ANOVA). Interestingly, the degree to which visual acuity was increased during monocular deprivation was age-dependent (one-way ANOVA with repeated measurements, F_3,30_ = 10,538, p<0,001). In general, visual acuity increased from 0,39 cyc/deg before deprivation to values of 0.50–0.53 cyc/deg on the seventh day after monocular deprivation (Fig. 3, PD25: 0.529±0.006, n = 9; PD95: 0.52±0.019, n = 17; PD130: 0.51±0.013, n = 7; PD215: 0.504±0.003, n = 14). While there was no difference between PD25 and PD95 animals, nor between PD130 and PD215 animals (p = 0.69 and p = 1, respectively, Bonferroni post-hoc), plasticity in PD95 animals differed significantly from both PD130 and PD215 animals (p<0.01/p<0.001, Bonferroni post-hoc). Additionally, values of PD25 animals were significantly different from those of PD215 animals (p<0.01).

## Discussion

The results of the present study show that ocular dominance plasticity in mice is most easily induced by monocular deprivation during the critical phase in early postnatal development (PD25), declines in young adults (PD95) and is absent in mature animals (PD130 and PD215). The enhancement of vision by monocular deprivation also declines after PD95, but much less dramatically.

### Ocular dominance plasticity in mice is age-dependent

While there are a number of recent studies reporting residual cortical plasticity in adult rodents, none has systematically compared ocular dominance plasticity after classical deprivation amblyopia (monocular deprivation) in animals of different age groups and most studies were concerned with animals younger than 100 days of age [19]. Our present results confirm previous observations in that 4 days of monocular deprivation are sufficient to induce a significant ocular dominance shift in PD25 animals [5], [6], [20], [21] and that the duration of monocular deprivation must be prolonged in order to induce a shift in young adult mice (PD95 [6], [8], [11], [22]). Surprisingly, already in the next age group, even 7 days of monocular deprivation did not induce a significant ocular dominance shift, and in PD215 animals even 14 days of monocular deprivation did not modify the ocular dominance of binocular visual cortex. While seemingly at odds with recent claims of “adult” plasticity being present throughout life, our observations are supported by numerous reports that also show little plasticity even after long-term adult monocular deprivation [12], [13], [23]–[25] and might even solve the recent discussion whether plasticity in rodent visual cortex is fundamentally different from other species such as cats and monkeys that show a clear age-dependence of ocular dominance plasticity. In cats, the critical period for a short, 10–12 days monocular deprivation ends after four months [4], [26], but long-term monocular deprivation for three months can still change ocular dominance in animals up to one year old but not older [27]. Thus ocular dominance plasticity ends at the completion of adolescence since cats are considered adult at around one year of age, as judged by e.g. body growth [28] or eye development [29]. In our hands, ocular dominance plasticity in mice also displays a clear critical period and an extended phase with reduced susceptibility for deprivation that is absent from animals older than 110 days which fits to the notion that plasticity does not end abruptly but rather declines gradually [26], [30]. These results are very important because they show that mice are not basically different from cats and monkeys (and presumably humans) in visual cortical plasticity which is a strong and absolutely necessary argument for the use of mice as model systems for disorders of the human visual system.

A thorough comparison of the literature reveals another interesting observation: plasticity in mouse visual cortex seems to be different from that in rats in which – at least up to now – no ocular dominance plasticity after monocular deprivation has been discovered in animals older than 55 days [13], [23], [31], [32]. Thus, given the lifespan of mice, these animals remain “plastic” for a much longer period of time (about 1/6 of their lifespan) compared to rats, cats and monkeys. Most importantly, however, ocular dominance plasticity terminates at a certain age in all of these animals.

Using sweep visual evoked potentials as a measure of cortical activity it was recently shown that 4 days of monocular deprivation resulted in an ocular dominance shift in mature (PD91-415) mice that was weaker than in juvenile animals [9]. In a later study, comparing the effects of 4 days of monocular deprivation among juvenile, fully adult (PD90–180) and mature (PD180–390) mice [10], visual evoked potential amplitude showed no significant difference among the age groups. However, the same study also found a maximal effect of monocular deprivation after as little as one day, whereas single unit recordings [5], conventional visual evoked potentials [6], [33], [34] and episodic optical imaging [8], [35] failed to detect any effect of monocular deprivation even after two or three days. In a recent meta-analysis of “adult” plasticity it was argued that not all changes accompanying monocular deprivation may be considered ocular dominance plasticity and that proper binocular vision rather reflects changes in visual cortex spike output that become limited with age [19]. This conclusion is totally consistent with the results of the present study that also indicate a clear end to ocular dominance plasticity in mouse visual cortex.

### Plasticity of visual acuity is age-dependent

Monocular deprivation improves visual acuity of the open eye while impairing vision in the closed eye; these changes are at least partly reversible after reopening the deprived eye [17], [36] and rely on the visual cortex [17]. In our behavioural experiments, the enhancement of vision by monocular deprivation was also age-dependent: Interocular plasticity in PD95 animals differed significantly from both PD130 and PD215 animals. In contrast to our optical imaging data, however, interocular plasticity was still present in animals older than 110 days. The dissociation of results (reduced versus absent plasticity) suggests that separate neural subsystems mediate the two forms of plasticity. In fact, using the optokinetic response, Prusky et al. [17] observed that the enhanced spatial frequency selectivity was restricted to the monocular visual field, notwithstanding the dependence of the plasticity on binocular interactions. In contrast, ocular dominance shifts after monocular deprivation were visualized in the binocular region of primary visual cortex. In addition, ocular dominance plasticity in animals beyond the critical period mostly happens in superficial cortical layers [7], [9], [27], while the enhancement of the optokinetic response involves the cortical control of the accessory optic system triggering the reflex [17], presumably from deep-layer efferents.

### Methodological considerations

Several recent studies have reported that certain anaesthetics, in particular barbiturates, may mask ocular dominance plasticity [9], [11], [14]. While halothane was the main anaesthetic in the present study, we gave a single dose of pentobarbital during tracheotomy. Although pentobarbital has a brain elimination half-life of approximately 40 min in mice [18], and thus should have been absent from our animals by the time we started recording maps, we wondered whether our failure to observe ocular dominance plasticity in mice older than 100 days might be due to residual barbiturates. We therefore repeated some of our experiments using urethane anaesthesia. In urethane-anaesthetized animals, 7 days of monocular deprivation did not induce a significant ocular dominance shift compared to controls. In addition, both optical imaging data and ocular dominance indices of these experiments were indistinguishable from those in animals with a single dose of pentobarbital. Our particular anaesthesia regime therefore did not mask ocular dominance plasticity in animals older than 100 days of age, a conclusion that is in line with a recent meta-analysis of ocular dominance plasticity also ruling out anaesthetics as a factor [19].

### Conclusion

The decline of ocular dominance plasticity as observed here with intrinsic signal optical imaging at around four months of age in mice suggests that there is something like an “extended critical period” for ocular dominance plasticity: the “classical” critical period in early postnatal animals followed by a period with reduced albeit present plasticity that terminates at around postnatal day 110. The mechanisms that regulate and terminate this period remain to be determined. A particular excitatory-inhibitory balance in the cortex is a likely candidate [19], but this has not yet been analysed in detail. There is accumulating evidence that a number of interventions can promote plasticity in adult rodents, including enzymatic degradation of the extracellular matrix [13], previous monocular deprivation of the same eye [8], environmental enrichment [37], visual deprivation [33], [38], stimulation of histone acetylation [34] and the antidepressant fluoxetine [32]. Since most of these therapeutic efforts have so far been applied only to animals younger than 100 days of age, it is an open question and clinically highly relevant - given the present results - whether they would also work in fully mature animals.
